# Nail dystrophy: a rare sign of sarcoidosis

**DOI:** 10.11604/pamj.2014.19.67.4988

**Published:** 2014-09-24

**Authors:** Nihal Bekkali, Mohammed Boui

**Affiliations:** 1 CHU Avicenne, Rabat, Maroc; 2 Hôpital Militaire Mohammed V, Rabat, Maroc

**Keywords:** Nail dystrophy, sarcoidosis, hyperkeratotic lesion

## Image in medicine

A 29- year- old woman presented to our clinic for subungueal hyperkeratotic lesion of the fourth left toenail. In 1997, she developed systemic sarcoidosis with lung and cutaneous lesions. The patient required gradually tapered systemic corticosteroids for 4 years and methotrexate for 1 year to control lung disease. A few months previously a hyperkeratotic verrucous lesion developed beneath the distal portion of the forth left toenail plate. There was no history of trauma. A biopsy specimen of the nail bed revealed epithelioid granulomas in the dermis without central necrosis. An X-ray of the feet showed severe osteolysis of the terminal phalanx of the forth left toenail. After 2 weeks application, once daily, of hight potency topical steroids, the subungueal hyperkeratosis rapidly improved without relapse after eight months. Nail dystrophy in sarcoidosis is rare. The most common nail changes described in sarcoidosis include thickening of the nail plates associated with fragility and longitudinal ridging, brown discoloration of the nail bed, convex nails and layering, splinter hemorrhage also occur. Such changes are usually associated with lupus pernio and cystic bone change of the phalanx. Our patient did not show lupus pernio like lesions, but radiological examination of the left foot showed bone cyst of the terminal phalanx. Treatment options for nail dystrophy in sarcoidosis include systemic treatment with 10mg/day of prednisone and 200mg/day of hydroxychlorochine sulphate, hight- potency topical steroids application once daily and steroid injections into the nail fold.

**Figure 1:**
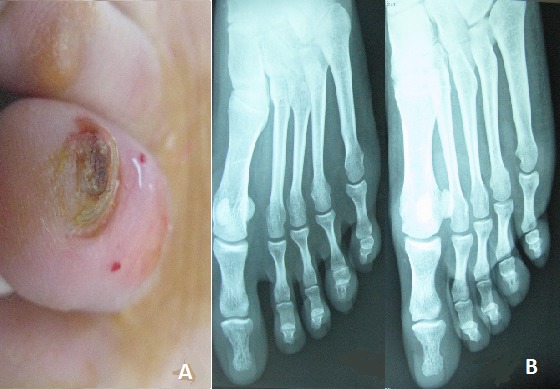
A) subungueal hyperkeratotic lesion of the toenail; B) bone cyst of the terminal phalanx

